# Relationship between aerobic performance and match running performance in elite soccer players including playing position and contextual factors

**DOI:** 10.5114/biolsport.2026.153311

**Published:** 2025-08-29

**Authors:** Jakub Kryściak, Tomas Maly, Maciej Tomczak, Toni Modric, James Malone, František Zahálka, Christian Clarup, Kirk Phillips, Marcin Andrzejewski

**Affiliations:** 1 Department of Physiology, Poznan University of Physical Education, Poznań, Poland; 2 Sport Research Center, Faculty of Physical Education and Sport, Charles University, Prague, Czechia; 3 Department of Performance, AC Sparta Praha, Prague, Czechia; 4 Department of Psychology, Poznań University of Physical Education, Poznan, Poland; 5 Faculty of Kinesiology, University of Split, Split, Croatia; 6 High Performance Sport Center, Croatian Olympic Committee, Zagreb, Croatia; 7 Malone Performance LTD, Liverpool, United Kingdom

**Keywords:** Soccer, Performance, Time–motion analysis, Physiology, GPS, Covered distance

## Abstract

This study aimed to examine the association between aerobic performance (AP) and match running performance (MRP) in elite soccer players when statistically controlling for playing position and contextual factors. AP was tested at the beginning of the season, including maximal oxygen uptake (${\dot{\text{V}}\text{O}}_{2\max}$), anaerobic threshold (AnT), maximal aerobic speed (MAS), and Yo-Yo Intermittent Recovery Test Level 2 (Yo-Yo IRT2) score. MRP was measured using GPS over a competitive half-season for a total of 216 match performances in elite soccer players, divided into central backs (CBs), full backs (FBs), central midfielders (CMs), wide midfielders (WMs), and strikers. The lowest AP measures were noted among the CBs, while the highest ${\dot{\text{V}}\text{O}}_{2\max}$, AnT, and MAS were recorded among the CMs, CMs and FBs, and FBs, respectively. The CBs had the lowest total distance (TD), high-speed running (HSR; 19.8–25.1 km · h^−1^) distance, and high-intensity running (HIR; > 19.8 km · h^−1^) distance; the CMs recorded the greatest TD; and the FBs and WMs covered the greatest HIR distance. Despite the differences in AP and MRP among the players, AP is correlated with MRP independently of the playing position and contextual factors. Higher AP measures were positively associated with the TD, and higher Yo-Yo IRT2 scores were also positively associated with the HSR and HIR distances. The strongest predictors for TD were ${\dot{\text{V}}\text{O}}_{2\max}$ and AnT. In conclusion, a higher AP, irrespective of playing position, makes it possible to achieve greater MRPs. This study emphasized the value of integrating AP metrics into individualized training and player role management in elite soccer.

## INTRODUCTION

In soccer, physical demands are typically evaluated based on match running performance (MRP). Approximately 85–95% of MRP is related to low-to-moderate-intensity activities (i.e., walking, jogging, and running) and only 5–15% to high-intensity activities (i.e., highspeed running [HSR] and sprinting [SPR]) [1]. The aerobic system is therefore the predominant source of energy during most matches [1, 2, 3, 4], making aerobic fitness crucial for football performance (i.e., aerobic performance [AP]). Specifically, adequate AP helps soccer players to maintain high-intensity actions at an appropriate level, delay the development of match fatigue, and accelerate postmatch recovery [2, 5].

Laboratory-based parameters, such as maximal oxygen uptake (${\dot{\text{V}}\text{O}}_{2\max}$) and anaerobic threshold (AnT), are typically used to evaluate AP. ${\dot{\text{V}}\text{O}}_{2\max}$ is the maximum volume of oxygen consumed during dynamic exercise to exhaustion involving large muscle groups [6] and typically normalized per kilogram of body mass (mL · kg^−1^ · min^−1^). The average ${\dot{\text{V}}\text{O}}_{2\max}$ among professional soccer players ranges from 55 to 65 mL · kg^−1^ · min^−1^ [3, 7, 8]. AnT is defined as the exercise load beyond which anaerobic glycolysis begins to dominate the metabolism of working muscles, and the production of lactate and hydrogen ions exceeds the ability to remove or neutralize them [9]. In professional soccer players, AnT typically occurs at running speeds ranging from 13.0 to 15.0 km · h^−1^ [3, 8].

Due to their field-based applicability [10, 11, 12], maximal aerobic speed (MAS) and the Yo-Yo test score have also become commonly used parameters for evaluating AP [10, 11, 12, 13]. MAS is defined as the lowest running velocity that elicits ${\dot{\text{V}}\text{O}}_{2\max}$ [14]. In professional soccer players, it usually occurs at speeds ranging from 4.3 to 4.9 m · s^−1^ [11, 12]. MAS can be determined either directly using laboratory exercise tests or indirectly using field tests. This metric is widely used by soccer practitioners for planning loads in soccer training [15, 16]. The Yo-Yo test has become markedly popular as a field-based equivalent to laboratory testing [10, 17, 18]. It is characterized by the intermittent nature of exercise and can be performed in two versions – Level 1 (Yo-Yo Intermittent Recovery Test [IRT] 1) and Level 2 (Yo-Yo IRT2) – which differ according to initial speed [10, 17]. The scores can be used to indirectly determine ${\dot{\text{V}}\text{O}}_{2\max}$.

AP indicators, including ${\dot{\text{V}}\text{O}}_{2\max}$, AnT, MAS, and the Yo-Yo test score, are significantly related to MRP in soccer [5, 7, 18, 19, 20, 21, 22]. Early research has identified a strong correlation between ${\dot{\text{V}}\text{O}}_{2\max}$ and the total distance (TD) covered during matches [23]. However, more recent studies have failed to establish a clear relationship between these variables [7, 8, 17, 24]. Modric et al. [8] found that AnT correlated with HSR, SPR, and high-intensity running (HIR) in wide players (e.g., full backs [FBs] and wingers) but not in central players (e.g., central defenders, central midfielders [CMs], and forwards), revealing this association as position-dependent. A significant relationship between MAS and TD during a match was first identified by Krustrup et al. [17] among elite male soccer players. Subsequent studies on youth soccer players demonstrated a moderate correlation (r = 0.45) between MAS and time spent in HIR [25] and between MAS and TD, specifically among forward players [26]. Additionally, Krustrup et al. [17] identified a significant positive correlation between the Yo-Yo IRT1 score and TD and HIR distance during soccer matches. Similarly, Bradley et al. [18] reported a strong relationship between the Yo-Yo IE2 score and both TD and HIR distance among soccer players. However, Bradley et al. [27] also reported this effect to be position-dependent.

The evident inconsistency in findings may be attributed to the different samples analysed or methodologies implemented. Firstly, some studies analysed data of soccer players in matches held 15–20 years ago [9, 17, 19, 23, 28, 29]. As longitudinal analyses of soccer matches have indicated that MRP has changed tremendously over the last decade [30], the findings from these studies are probably limited in current application. Secondly, studies rarely considered match-related factors that can significantly affect MRP or AP, such as playing position, match outcome, match location, and opponent quality. To the best of our knowledge, only two studies have shown a position-specific relationship between MRP and AP [7, 8], and no study has yet statistically controlled for playing position and any other factors that can influence this relationship (contextual factors). Thirdly, studies investigating the position-specific relationship between MRP and AP exclusively implemented simplistic statistical approaches [7, 8], which are not able to control the natural variability in MRP [31, 32, 33], as opposed to more advanced statistical techniques such as the linear mixed model (LMM). As a result, the true effect of AP on MRP may have been obscured, possibly leading to potentially misleading findings. Consequently, actual knowledge on the relation between AP and MRP remains limited.

Taking into account that these methodological limitations represent a significant gap in the current literature, this study aimed to: i) evaluate the relationship between AP and MRP when statistically controlling for specific playing position, selected contextual factors and individual variability of soccer players, ii) identify which routinely measured AP indicators show the strongest predictive value for MRP variables.

## MATERIALS AND METHODS

### Design

Players competing at the highest level (current national champion) in one of the central European leagues over one half-season were observed. MRP was measured using portable global positioning system (GPS) units in all domestic league and European cup matches (including qualification) from July to December 2023. MRP for at least 75 min from the start of matches was analysed [32, 34]. AP indicators (independent variables) were examined using laboratory (ergospirometric exercise test to volitional exhaustion) and field tests (Yo-Yo IRT2) at the beginning of the pre-season period. The relationship between MRP and AP was analysed first, followed by additional consideration of contextual factors such as match location (H/A) or competition level (EC/DL).

### Participants

The MRP of 21 professional players (mean ± SD, age: 25.2 ± 3.5 years; height: 184.1 ± 6.1 cm; body mass: 81.0 ± 6.0 kg) playing in the same soccer team was analysed. Only outfield players were observed, and they were divided into the following playing positions: central backs (CBs; n = 6), full backs (FBs; n = 5), central midfielders (CMs; n = 3), wide midfielders (WMs; n = 4), and strikers (STs; n = 3). The categorization of players into specific playing positions was based on the system of play (1-5-4-1) used in the matches analysed, which includes in the defensive formation three central backs and two full backs, in the midfield formation two central midfielders and two wide midfielders, and in the attack formation one striker. Players were observed in a total of 29 matches (19 matches in domestic league and 10 matches in European competition), resulting in 216 match performances (CBs: n = 77; FBs: n = 43; CMs: n = 41; WMs: n = 27; STs: n = 28). All players were fully informed about the study objectives and signed an informed consent form agreeing to participate in the study. The typical team microcycle for this period had a double peak (two matches in a week) (Table 1).

**TABLE 1:** Overview of the weekly training/match in-season microcycle in relation to a match day

Day	Field-based training	Gym training	Match
MD +1	Recovery^a^, top-up (60 min)^b^	30 min^a,b^	
MD -2	60 min		
MD -1	60 min		
MD		20 min^c^	90 min
MD +1	Recovery^a^, top-up (60 min)^b^	30 min^a,b^	
MD -1	60 min		
MD		20 min^c^	90 min

MD, match day;

a recovery and gym session for players who played more than 45 min;

b in-field-based session for players who either did not appear in line-up or played less than 45 min;

c prime prematch session aiming to use the post-activation potentiation effect.

Measurements were performed according to the Declaration of Helsinki and the ethical standards in sport and exercise science research described by Harriss et al. [35]. The study followed valid principles, regulations, and international guidelines for research involving human participants and was approved by the institutional ethics committee (Ethical Commitee of Faculty of Physical Education and Sport, Charles University, Prague, no. 068/2024).

### Procedures

#### MRP assessment

GPS units (SONRA, StatSports, Newry, Northern Ireland) were used to measure the performance of players. The scanning device had a gyroscope (100 Hz), an accelerometer scanning in the x-, y-, and z-axes (100 Hz), and a magnetometer (10 Hz). The GPS technology is valid and reliable for monitoring external workloads during competitions [36]. All devices were activated 30 min before data collection to connect and synchronize the GPS signal from satellites. Players wore the same unit over the whole season to avoid inter-unit error [37], although high inter-unit reliability was reported by Beato and de Keijzer [38].

MRP was assessed based on the TD covered during matches and distance covered in three speed categories: HSR (19.8–25.1 km · h^−1^), SPR (≥ 25.2 km · h^−1^), and HIR (> 19.8 km · h^−1^). As MRP was measured in players playing for at least 75 min (cut-off point) from the start of matches, all indicators were presented in relative units, converted per minute.

### AP assessment

All AP tests were performed at the beginning of the pre-season period. Laboratory AP measures were assessed during graded exercise tests (GXTs) to volitional exhaustion and included ${\dot{\text{V}}\text{O}}_{2\max}$, AnT, and MAS. All tests were conducted from 9:00 a.m. to noon, 24 h after the last practice session, to minimize the effect of fatigue. The GXTs were conducted in an air-conditioned laboratory room (air temperature: 19–22°C, relative humidity: approximately 50%) 2 h after a light breakfast. The tests started by having players run at 11 km · h^−1^ on a flat treadmill. After 3 min, with no change in belt speed, the incline of the treadmill was increased to 5% for 1 min. The treadmill speed was then increased by 1 km · h^−1^ every minute until volitional exhaustion. Expired gases were collected continuously throughout the GXTs using the Cortex MetaLyzer 3B gas analyser (Cortex Biophysik, Leipzig, Germany). ${\dot{\text{V}}\text{O}}_{2\max}$ (mL · kg^−1^ · min^−1^) was recorded and considered achieved when the test met at least three of the following criteria: i) a plateau in ${\dot{\text{V}}\text{O}}_{2}$ with increasing workload, ii) a respiratory gas exchange ratio of > 1.15, iii) an HR within 5 beats · min^−1^ of the age-predicted maximal HR, and iv) an inability to continue running despite verbal encouragement [7]. MAS was defined as the lowest running velocity that elicited ${\dot{\text{V}}\text{O}}_{2\max}$ [14].

AnT was determined using the ventilatory threshold method based on ventilation (VE) plots and ventilatory equivalents (VE/${\dot{\text{V}}\text{O}}_{2}$ and VE/${\dot{\text{V}}\text{CO}}_{2}$) as functional parameters of oxygen uptake and time. The criteria used were as follows: i) an increase in VE/${\dot{\text{V}}\text{O}}_{2}$ with a concomitant increase in VE/${\dot{\text{V}}\text{CO}}_{2}$ and ii) second non-linear increases in VE plots [39].

The field-based Yo-Yo IRT2 was conducted after all players were fully familiarized with the test procedure. The test started at 13 km · h^−1^ and consisted of 2 × 20-m shuttle runs at increasing velocities with 10 s of active recovery between shuttles [40]. The running pace was dictated by an audible signal. Stepwise speed increments followed until participants either failed to reach the line (objectively evaluated by two experienced researchers) twice or felt too exhausted to continue at the required speed (subjectively evaluated). Before the Yo-Yo test, all players conducted a standardized warm-up. They performed the test on an official natural grass field, wearing footwear that met the standards required by the sport. The AP values are presented in Table 2.

**TABLE 2:** Aerobic performance values

Variable	Mean ± SD
${\dot{\text{V}}\text{O}}_{2\max}$ (mL · kg^−1^ · min^−1^)	60.0 ± 2.6
AnT (km · h^−1^)	14.3 ± 0.8
MAS (km · h^−1^)	16.9 ± 0.7
Yo-Yo IRT2 score (m)	1283 ± 177

${\dot{\text{V}}\text{O}}_{2\max}$, maximal oxygen uptake; AnT, anaerobic threshold; MAS, maximal aerobic speed; Yo-Yo IRT2, Yo-Yo Intermittent Recovery Test level 2

### Statistical analysis

The differences between AP and MRP among soccer players in different positions were analysed first. Due to unequal variances combined with different numbers of observations among the groups, the non-parametric Kruskal–Wallis H test and Dunn’s post-hoc test were used to compare the variables. Epsilon squared (ε^2^) was used as a measure of the effect size. The distributions of all observations suggested that their skewness and kurtosis indices do not indicate large normality violations (skewness and kurtosis below 1). The skewness index was 1.45 only for the SPR distance, and the kurtosis index was 2.53 for the SPR distance and −1.33 for the ${\dot{\text{V}}\text{O}}_{2\max}$. However, the non-parametric Spearman correlation coefficients did not strongly differ from the Pearson correlation coefficients. Hence, the Pearson correlation coefficients were used to assess the strength of the relationship between AP and MRP. This analysis was conducted in the Statistica software (version 13.3).

Linear mixed models were used to assess the relationship between AP and MRP when controlling for individual players, positions, competition levels (EC/DL), and match locations (H/A) and considering the interaction between position and AP. Visual analysis of the distributions of the residuals and the Kolmogorov–Smirnov test (mixed models module; Jamovi software) generally did not indicate significant violations of the normality conditions. MRP was used as the dependent variable and AP as the main independent variable. Position, competition level, and match location were considered additional (control) independent variables. The main and additional independent variables were modelled as fixed effects and the player effect as a random effect (random intercept). Due to the relatively high correlation coefficients between the AP parameters and given the number of observations analysed, models were estimated separately for each parameter. Initially, models without interactions were estimated, and interaction effects between position and individual AP parameters were added. For significant relationships between the main independent variables (AP variables) and the dependent variables (MRP variables), as indicators of effect size standardized coefficients are presented, which can be interpreted as regression coefficients, where values of 0.10–0.29 are defined as small, values of 0.30–0.49 as medium and values of 0.50 or more as large [41]. The aim of the models estimated first was to assess the relationship between AP and MRP under statistical control for the additional (control) variables (competition level, match location, and position). The main aim of the models consequently estimated was to evaluate the interaction effects of the AP parameters with position on MRP. The analysis was performed on the centred variables, with simple coding used for the qualitative variables interacting with the AP parameters. The CB position was used as the reference position because of the deviating requirements on this position from the other positions relative to MRP and AP. This analysis was performed in Jamovi (version 2.3.28).

## RESULTS

### AP and MRP differences according to playing position

Among the players, the lowest ${\dot{\text{V}}\text{O}}_{2\max}$ was observed in the CBs, with significant post-hoc differences. Conversely, the highest ${\dot{\text{V}}\text{O}}_{2\max}$ was recorded in the CMs, also with significant differences. The CBs had a significantly lower AnT than the other players. Additionally, the FBs and CMs had a higher AnT than the WMs and STs. The MAS and Yo-Yo IRT2 score were significantly higher among the FBs than among the other players. A lower Yo-Yo IRT2 score was observed in the CBs than in the CMs (Table 3).

**TABLE 3:** Descriptive statistics of MRP and AP and differences between players in soccer-specific playing positions

		CB (n = 77) Mean rank Mean (SD)	FB (n = 43) Mean rank Mean (SD)	CM (n = 41) Mean rank Mean (SD)	WM (n = 27) Mean rank Mean (SD)	ST (n = 28) Mean rank Mean (SD)	H	p	ε^2^
**MRP**	TD (m/min)	62.1^WM,CM,FB,ST^ 104.4 (6.9)	127.7^CB^ 112.1 (8.0)	161.2^CB,WM^ 115.5 (4.4)	113.7^CB,CM^ 110.4 (3.5)	124.4^CB^ 111.6 (6.3)	77.69	< 0.001	0.36

	HSR distance (m/min)	46.7^WM,CM,FB,ST^ 4.6 (1.1)	151.9^CB^ 8.0 (2.0)	125.2^CB^ 6.8 (1.2)	167.6^CB^ 8.1 (1.2)	130.4^CB^ 6.9 (1.3)	126.59	< 0.001	0.59

	SPR distance (m/min)	60.6^WM,FB,ST^ 1.2 (0.6)	153.3^CB,CM^ 2.7 (1.1)	84.0^FB,WM,ST^ 1.4 (0.6)	167.4^CB,CM^ 3.4 (1.7)	150.4^CB,CM^ 2.7 (1.1)	110.26	< 0.001	0.51

	HIR distance (m/min)	46.6^WM,CM,FB,ST^ 5.7 (1.4)	155.1^CB,CM^ 10.7 (3.0)	111.2^CB,FB,WM^ 8.3 (1.5)	171.4^CB,CM^ 11.6 (2.7)	142.7^CB^ 9.7 (1.8)	135.21	< 0.001	0.63

**AP**	${\dot{\text{V}}\text{O}}_{2\max}$ (mL · kg^−1^ · min^−1^)	52.8^WM,CM,FB,ST^ 57.5 (1.5)	124.1^CB,CM^ 61.1 (2.8)	189.6^CB,FB,WM,ST^ 63.3 (0.6)	132.8^CB,CM^ 61.1 (0.9)	95.6^CB,CM^ 59.7 (1.3)	138.87	< 0.001	0.65

	AnT (km · h^−1^)	46.4^WM,CM,FB,ST^ 13.4 (0.6)	167.0^CB,WM,ST^ 15.1 (0.8)	162.9^CB,WM,ST^ 14.9 (0.2)	100.7^CB,FB,CM^ 14.3 (0.3)	117.2^CB,FB,CM^ 14.5 (0.4)	146.45	< 0.001	0.68

	MAS (km · h^−1^)	91.4^FB^ 16.8 (0.6)	163.2^CB,WM,CM,ST^ 17.6 (0.8)	107.2^FB^ 17.1 (0.1)	78.8^FB^ 16.6 (0.5)	102.1^FB^ 17.0 (0.5)	48.46	< 0.001	0.22

	Yo-Yo IRT2 score (m)	80.1^CM,FB^	173.8^CB,WM,CM,ST^ 1196.4 (151.4) 1476.3 (193.6)	120.2^CB,FB^ 1296.6 (52.9)	99.8^FB^	77.5^FB^ 1277.0 (101.6) 1217.1 (106.9)	72.97	< 0.001	0.34

Superscripted letters indicate significant post-hoc differences (p < 0.05) when compared to specific playing positions.

ε^2^, indicator of the effect size; CB, central back; FB, full back; CM, central midfielder; WM, wide midfielder; ST, striker; MRP, match running performance; AP, aerobic performance; TD, total distance; HSR, high-speed running; SPR, sprinting; HIR, high-intensity running; ${\dot{\text{V}}\text{O}}_{2\max}$, maximal oxygen uptake; AnT, anaerobic threshold; MAS, maximal aerobic speed; Yo-Yo IRT2, Yo-Yo Intermittent Recovery Test level 2

The CBs covered significantly lower TD, HSR distance, and HIR distance than the other players. A significantly greater TD was recorded in the CMs than in the WMs. The CBs and CMs demonstrated a lower SPR distance than the other players. Additionally, the CMs covered a lower HIR distance than the FBs and WMs (Table 3).

### General associations between AP and MRP

Positive correlations were observed between the AnT and Yo-Yo IRT2 score and all MRP indicators. Additionally, relatively strong correlations were noted between the ${\dot{\text{V}}\text{O}}_{2\max}$ and TD, HSR distance, and HIR distance and moderate correlations between the ${\dot{\text{V}}\text{O}}_{2\max}$ and SPR distance. The MAS significantly correlated with the TD, HSR distance, and HIR distance. No significant relationship was observed between the MAS and SPR distance (p = 0.41) (Table 4).

**TABLE 4:** Pearson correlation coefficients between aerobic performance and match running performance indicators without disaggregation by soccer-specific playing positions

	TD (n = 216)	HSR distance (n = 216)	SPR distance (n = 216)	HIR distance (n = 216)
** ${\dot{\text{V}}\text{O}}_{2\max}$ **	0.68 (p < 0.001)	0.52 (p < 0.001)	0.15 (p = 0.032)	0.41 (p < 0.001)
**AnT**	0.66 (p < 0.001)	0.60 (p < 0.001)	0.27 (p < 0.001)	0.51 (p < 0.001)
**MAS**	0.49 (p < 0.001)	0.35 (p < 0.001)	0.06 (p = 0.413)	0.25 (p < 0.001)
**Yo-Yo IRT2 score**	0.43 (p < 0.001)	0.54 (p < 0.001)	0.35 (p < 0.001)	0.51 (p < 0.001)

TD, total distance; HSR, high-speed running; SPR, sprinting; HIR, high-intensity running; ${\dot{\text{V}}\text{O}}_{2\max}$, maximal oxygen uptake; AnT, anaerobic threshold; MAS, maximal aerobic speed; Yo-Yo IRT2, Yo-Yo Intermittent Recovery Test level 2

### Relationships between AP and MRP interacting with playing position and contextual factors

In the models estimated first, there was a significant positive association between the ${\dot{\text{V}}\text{O}}_{2\max}$ (Table 5), AnT (Table 6), MAS (Table 7), and TD when statistically controlling for the playing position, match location (H/A), and competition level (EC/DL). For an increase in the ${\dot{\text{V}}\text{O}}_{2\max}$ by every 1 mL · kg^−1^ · min^−1^, AnT by every 1 km · h^−1^, and MAS by every 1 km · h^−1^, the TD increased on average by 1.920, 5.601, and 5.419 m/min, respectively. The standardized coefficients for the relationship between ${\dot{\text{V}}\text{O}}_{2\max}$, AnT, MAS and TD were β = 0.69, 0.67 and 0.45, respectively. In contrast, these AP indicators showed no significant associations with the HSR, SPR, and HIR distances. For the Yo-Yo IRT2 score, there were significant positive relationships with the TD, HSR distance, and HIR distance (Table 8).

**TABLE 5:** Relationships between ${\dot{\text{V}}\text{O}}_{2\max}$ and MRP with statistical controls for player position, match location, competition level and interaction effects of ${\dot{\text{V}}\text{O}}_{2\max}$ with player position.

	TD		HSR		SPR		HIR	
*Fixed effects*	b	SE	b	SE	b	SE	b	SE

Intercept	109.954^***^	0.533	6.821^***^	0.201	2.374^***^	0.202	9.195^***^	0.380
Pos l (1–2)	-0.888	1.822	2.832^***^	0.665	2.206^**^	0.660	5.043^***^	1.246
Pos 2 (3–2)	0.378	2.181	1.368	0.825	0.636	0.832	2.014	1.563
Pos 3 (4–2)	0.619	1.653	2.808^***^	0.607	1.804^**^	0.604	4.617^***^	1.140
Pos 4 (5–2)	3.023	1.700	2.111^**^	0.636	1.978^**^	0.635	4.109^**^	1.197
${\dot{\text{V}}\text{O}}_{2\max}$	1.920 ^***^	0.285	0.173	0.106	-0.068	0.105	0.105	0.198
H/A	2.544^***^	0.688	0.406^*^	0.159	0.244^**^	0.091	0.644^**^	0.211
EC/DL	3.225^***^	0.775	0.425^*^	0.182	-0.013	0.105	0.399	0.244

*Random effects*	Variance		Variance		Variance		Variance	

Player ID	2.499		0.605		0.725		2.462	
Residual	24.620		1.301		0.427		2.290	

R^2^ marginal	0.541		0.550		0.395		0.523	
R^2^ conditional	0.583		0.693		0.776		0.777	
AIC	1330.297		715.702		498.600		852.805	
Models with interactions								
	**TD**		**HSR**		**SPR**		**HIR**	
*Fixed effects*	b	SE	b	SE	b	SE	b	SE
Intercept	111.357^***^	1.357	6.990^***^	0.598	2.244^**^	0.613	9.230^***^	1.160
Pos l (1–2)	-1.505	2.504	3.266^**^	0.999	1.929	0.987	5.179^*^	1.886
Pos 2 (3–2)	2.981	6.257	2.744	2.786	0.364	2.863	3.101	5.421
Pos 3 (4–2)	-1.118	1.941	2.986^**^	0.803	1.664	0.800	4.651^*^	1.526
Pos 4 (5–2)	0.808	2.168	2.155^*^	0.939	1.437	0.952	3.590	1.809
${\dot{\text{V}}\text{O}}_{2\max}$	1.418^*^	0.521	0.020	0.221	-0.077	0.224	-0.054	0.425
H/A	2.531^***^	0.690	0.405^*^	0.159	0.245^**^	0.091	0.645^**^	0.211
EC/DL	3.192^***^	0.777	0.414^*^	0.183	-0.014	0.106	0.391	0.244
Posl^*^${\dot{\text{V}}\text{O}}_{2\max}$	-1.923	1.542	-0.099	0.613	0.110	0.608	0.023	1.161
Pos2^*^${\dot{\text{V}}\text{O}}_{2\max}$	-2.132	1.945	-0.256	0.878	-0.004	0.905	-0.258	1.712
Pos3^*^${\dot{\text{V}}\text{O}}_{2\max}$	-0.815	0.673	0.208	0.289	-0.007	0.292	0.202	0.555
Pos4^*^${\dot{\text{V}}\text{O}}_{2\max}$	-1.377	1.049	-0.088	0.411	-0.410	0.404	-0.503	0.773
*Random effects*	Variance		Variance		Variance		Variance	
Player ID	2.234		0.753		0.878		3.080	
Residual	24.778		1.303		0.427		2.292	
R^2^ marginal	0.553		0.532		0.361		0.491	
R^2^ conditional	0.590		0.703		0.791		0.783	
AIC	1331.821		721.414		503.960		858.760	

MRP, match running performance; ${\dot{\text{V}}\text{O}}_{2\max}$, maximal oxygen uptake; TD, total distance; HSR, high-speed running; SPR, sprinting; HIR, high-intensity running; H/A, home/away; EC/DL, European cup/domestic league;

*** p<0.001,

** p<0.01,

* p<0.05.

**TABLE 6:** Relationships between AnT and MRP with statistical controls for player position, match location, competition level and interaction effects of AnT with player position.

TD	TD		HSR		SPR		HIR
*Fixed effects*	b	SE	b	SE	b	SE	b	SE

Intercept	109.995^***^	0.570	6.812^***^	0.194	2.362^***^	0.204	9.176^***^	0.373
Pos l (1–2)	0.973	1.807	2.903^***^	0.604	2.046^**^	0.630	4.952^***^	1.152
Pos 2 (3–2)	2.928	2.081	1.437	0.718	0.370	0.765	1.820	1.391
Pos 3 (4–2)	-2.058	2.038	2.425^**^	0.671	1.714^*^	0.691	4.161^**^	1.269
Pos 4 (5–2)	1.236	1.924	1.853^*^	0.645	1.959^*^	0.670	3.834^**^	1.229
AnT	5.601^***^	0.907	0.611	0.305	-0.069	0.319	0.543	0.584
H/A	2.407^***^	0.688	0.401^*^	0.159	0.244^**^	0.091	0.642^**^	0.211
EC/DL	3.006^***^	0.777	0.419^*^	0.182	-0.011	0.105	0.398	0.244

*Random effects*	Variance		Variance		Variance		Variance	

Player ID	3.238		0.552		0.744		2.362	
Residual	24.603		1.300		0.427		2.290	

R^2^ marginal	0.531		0.564		0.397		0.537	
R^2^ conditional	0.585		0.694		0.780		0.772	
AIC	1332.969		714.166		499.113		852.018	

Models with interactions								
	**TD**		**HSR**		**SPR**		**HIR**	
*Fixed effects*	b	SE	b	SE	b	SE	b	SE

Intercept	109.360^***^	1.303	6.616^***^	0.412	2.153^***^	0.410	8.775^***^	0.771
Pos l (1–2)	1.239	2.191	3.353^***^	0.676	2.097^**^	0.655	5.437^***^	1.243
Pos 2 (3–2)	0.316	5.959	1.727	1.897	-0.177	1.899	1.547	3.569
Pos 3 (4–2)	-2.207	2.274	2.654^**^	0.696	1.764^*^	0.670	4.444^**^	1.274
Pos 4 (5–2)	1.475	2.298	2.333^**^	0.714	1.994^*^	0.698	4.323^**^	1.321
AnT	5.407^*^	2.231	0.919	0.699	0.619	0.692	1.532	1.305
H/A	2.423^***^	0.691	0.403^*^	0.159	0.246^**^	0.091	0.645^**^	0.211
EC/DL	2.994^***^	0.785	0.402^*^	0.182	-0.021	0.105	0.374	0.244
Pos l^*^AnT	-4.722	5.807	2.747	1.789	3.802	1.741	6.535	3.297
Pos 2^*^AnT	4.707	9.005	0.934	2.872	1.244	2.879	2.173	5.410
Pos 3^*^AnT	0.717	2.107	1.016	0.660	0.295	0.650	1.295	1.227
Pos 4^*^AnT	-0.535	3.630	-0.060	1.077	-1.068	1.012	-1.151	1.935

*Random effects*	Variance		Variance		Variance		Variance	

Player ID	4.088		0.523		0.604		2.064	
Residual	24.722		1.302		0.427		2.291	

R^2^ marginal	0.525		0.572		0.448		0.563	
R^2^ conditional	0.592		0.695		0.771		0.770	
AIC	1338.704		715.250		496.667		851.195	

MRP, match running performance; AnT, anaerobic threshold; TD, total distance; HSR, high-speed running; SPR, sprinting; HIR, highintensity running; H/A, home/away; EC/DL, European cup/domestic league

**TABLE 7:** Relationships between MAS and MRP with statistical controls for player position, match location, competition level and interaction effects of MAS with player position.

	TD		HSR		SPR		HIR	
*Fixed effects*	b	SE	b	SE	b	SE	b	SE

Intercept	110.848^***^	0.623	6.905^***^	0.194	2.345^***^	0.203	9.253^***^	0.375
Pos l (1–2)	6.186^**^	1.818	3.455^***^	0.559	1.975^**^	0.577	5.424^***^	1.071
Pos 2 (3–2)	9.137^***^	1.852	2.099^**^	0.591	0.325	0.630	2.429	1.160
Pos 3 (4–2)	2.566	1.804	2.906^***^	0.556	1.716^**^	0.575	4.627^***^	1.067
Pos 4 (5–2)	5.561^*^	1.949	2.312^**^	0.607	1.904^**^	0.631	4.233^**^	1.169
MAS	5.419^***^	1.023	0.585	0.312	-0.150	0.321	0.429	0.596
H/A	2.478^***^	0.690	0.404^*^	0.159	0.244^**^	0.091	0.643^**^	0.211
EC/DL	3.034^***^	0.783	0.421^*^	0.182	-0.012	0.105	0.398	0.244

*Random effects*	Variance		Variance		Variance		Variance	

Player ID	4.611		0.566		0.735		2.417	
Residual	24.677		1.301		0.427		2.291	

R^2^ marginal	0.491		0.549		0.399		0.523	
R^2^ conditional	0.571		0.686		0.779		0.768	
AIC	1337.830		714.708		498.877		852.474	

Models with interactions								

**TD**		**HSR**		**SPR**		**HIR**	
*Fixed effects*	b	SE	b	SE	b	SE	b	SE

Intercept	110.219^***^	0.716	6.730^***^	0.224	2.262^***^	0.264	8.997^***^	0.466
Pos l (1–2)	5.068^*^	2.123	3.115^***^	0.653	1.884^*^	0.755	5.007^**^	1.340
Pos 2 (3–2)	8.479^**^	2.162	1.959^*^	0.694	0.122	0.836	2.084	1.470
Pos 3 (4–2)	1.946	1.877	2.676^***^	0.580	1.633^*^	0.672	4.325^**^	1.192
Pos 4 (5–2)	5.641^*^	2.056	2.183^**^	0.653	1.762^*^	0.779	3.938^*^	1.372
MAS	6.425^*^	2.309	0.660	0.733	0.134	0.875	0.800	1.541
H/A	2.466^***^	0.691	0.404^*^	0.159	0.244^**^	0.091	0.642^**^	0.211
EC/DL	2.929^***^	0.794	0.400^*^	0.184	-0.016	0.106	0.381	0.245
Pos l*MAS	-2.984	3.385	-0.962	1.040	-0.281	1.203	-1.203	2.134
Pos 2*MAS	8.995	10.77	1.497	3.443	1.700	4.142	3.188	7.283
Pos 3*MAS	2.389	2.463	0.583	0.771	0.068	0.907	0.652	1.602
Pos 4*MAS	0.095	3.329	-0.604	0.992	-0.518	1.113	-1.112	1.988

*Random effects*	Variance		Variance		Variance		Variance	

Player ID	4.707		0.582		0.964		2.899	
Residual	24.701		1.303		0.427		2.292	

R^2^ marginal	0.501		0.562		0.357		0.510	
R^2^ conditional	0.581		0.697		0.803		0.784	
AIC	1340.402		717.101		505.823		857.627	

MRP, match running performance; MAS, maximal aerobic speed; TD, total distance; HSR, high-speed running; SPR, sprinting; HIR, high-intensity running; H/A, home/away; EC/DL, European cup/domestic league

**TABLE 8:** Relationships between YoYo IRT2 and MRP with statistical controls for player position, match location, competition level and interaction effects of YoYo IRT2 with player position.

	TD		HSR		SPR		HIR	
*Fixed effects*	b	SE	b	SE	b	SE	b	SE

Intercept	110.592^***^	0.785	6.864^***^	0.150	2.360^***^	0.196	9.228^***^	0.319
Pos l (1–2)	4.082	2.306	3.099^***^	0.443	1.889^**^	0.570	4.973^***^	0.931
Pos 2 (3–2)	8.886^**^	2.413	1.873^**^	0.453	0.135	0.620	2.010	0.997
Pos 3 (4–2)	1.233	2.497	2.266^***^	0.478	1.294	0.620	3.567^**^	1.010
Pos 4 (5–2)	5.952^*^	2.464	2.349^***^	0.469	1.951^**^	0.615	4.323^***^	1.002
YOYO	0.019^**^	0.005	0.004^**^	0.001	0.001	0.001	0.005^*^	0.002
H/A	2.449^***^	0.691	0.410^*^	0.159	0.244^**^	0.091	0.645^**^	0.211
EC/DL	3.011^***^	0.792	0.448^*^	0.180	-0.008	0.105	0.416	0.243

*Random effects*	Variance		Variance		Variance		Variance	

Player ID	8.957		0.281		0.694		1.690	
Residual	24.714		1.302		0.427		2.293	

R^2^ marginal	0.432		0.612		0.431		0.592	
R^2^ conditional	0.583		0.681		0.783		0.765	
AIC	1347.693		704.162		497.813		846.047	

Models with interactions									
**TD**	**HSR**		**SPR**		**HIR**	

*Fixed effects*	b	SE	b	SE	b	SE	b	SE
Intercept	110.248^***^	0.977	6.711^***^	0.166	2.287^***^	0.217	8.999^***^	0.354
Pos l (1–2)	4.751	2.606	3.270^***^	0.448	2.011^**^	0.571	5.263^***^	0.941
Pos 2 (3–2)	9.386^**^	2.837	1.958^**^	0.470	0.141	0.642	2.093	1.039
Pos 3 (4–2)	0.920	2.801	2.187^***^	0.482	1.450^*^	0.614	3.663^**^	1.011
Pos 4 (5–2)	7.020	3.221	2.115^**^	0.541	1.684^*^	0.722	3.785^**^	1.174
YOYO	0.019	0.010	0.004^*^	0.002	0.003	0.002	0.007	0.004
H/A	2.427^***^	0.693	0.410^*^	0.159	0.245^**^	0.091	0.648^**^	0.211
EC/DL	3.040^***^	0.801	0.418^*^	0.183	-0.016	0.106	0.386	0.244
Pos l* YOYO	-0.003	0.022	0.005	0.004	0.010	0.005	0.015	0.008
Pos 2* YOYO	0.020	0.042	0.004	0.007	0.004	0.010	0.009	0.016
Pos 3* YOYO	0.015	0.014	0.003	0.002	0.000	0.003	0.003	0.005
Pos 4* YOYO	0.012	0.019	-0.003	0.003	-0.002	0.004	-0.004	0.007

*Random effects*	Variance		Variance		Variance		Variance	

Player ID	10.750		0.250		0.613		1.517	
Residual	24.757		1.302		0.427		2.291	

R^2^ marginal	0.416		0.621		0.471		0.610	
R^2^ conditional	0.593		0.682		0.783		0.765	
AIC	1352.702		704.048		496.871		845.536	

MRP, match running performance; YOYO, Yo-Yo Intermittent Recovery Test level 2; TD, total distance; HSR, high-speed running; SPR, sprinting; HIR, high-intensity running; H/A, home/away; EC/DL, European cup/domestic league

For every 1 m increase in the Yo-Yo IRT2 score, the TD increased on average by 0.019 m/min, HSR distance by 0.004 m/min, and HIR distance by 0.005 m/min. The standardized coefficients for the relationship between Yo-Yo IRT2 score and TD, HSR distance, and HIR distance were β = 0.43, 0.35 and 0.30, respectively. Additionally, the match location was systematically related to MRP when controlling for the other variables. The players had significantly higher MRP indicators in home matches than in away matches (TD: 2.5 m/min, HSR distance: 0.4 m/min, SPR distance: 0.24 m/min, and HIR distance: 0.65 m/min). Significant relationships were also noted between the competition level and TD and HSR distance, with the players having significantly higher MRP values in European competitions (TD: 3.0 m/min, HSR distance: 0.42 m/min). In some cases, MRP differed between the CBs and other players (Tables 5–8). Adding interaction effects generally did not strongly affect the model fit. There were no significant interaction effects of the AP indicators with the player position, indicating a relatively similar relationship between the AP and MRP indicators in all positions studied compared to the control position (CB) (interaction models: Tables 5–8).

## DISCUSSION

This was the first study designed to evaluate the relationship between AP and MRP while statistically controlling for playing position and contextual factors, and at the same time accounting for players’ individual variability in MRP. The main finding is that despite the differences in AP and MRP among the players studied, AP is related to MRP independently of playing position and contextual factors. A higher AP was positively associated with the TD covered during matches, and a higher Yo-Yo IRT2 score also positively associated with the HSR and HIR distances. These findings demonstrated that, regardless of position, enhancing AP may help players sustain greater match workloads, making it one of the key targets for conditioning programmes.

Differences in AP and MRP according to soccer-specific playing positions were analysed. The lowest AP, including the ${\dot{\text{V}}\text{O}}_{2\max}$, AnT, and Yo-Yo IRT2 score, was observed in the CBs. Conversely, the CMs had the highest ${\dot{\text{V}}\text{O}}_{2\max}$. The FBs had the highest MAS and Yo-Yo IRT2 score and showed a high AnT, similar to the CMs. In contrast, the AP indicators did not significantly differ between the WMs and STs. Our results confirm the previously found position-specific differences in AP [8, 9, 22, 42, 43]. Variations in AP at individual positions can result from position-specific conditional training programmes, match demands, and anthropometric characteristics [42, 43]. CBs generally cover the shortest TD, HSR distance, and SPR distance [8, 18, 29, 44], which is related to the fact that CBs are least involved in offensive actions, and their movement into the opponents’ penalty area is primarily related to corner kicks and free kicks. The longest TD during a match is usually noted among CMs and FBs [8, 9, 18, 44]. This is because these players have both defensive and offensive tasks, consequently involving frequent movements up and down the field. The TD results obtained in our study are consistent with the above-mentioned data, with the shortest and longest TDs recorded in the CBs and CMs, respectively. The CBs also showed the shortest HSR distance, similar to previous reports [8, 9, 18, 29, 44]. In line with previous studies [18, 44], we observed significantly greater SPR distances among the side players (FBs and WMs) and STs than among the CBs and CMs. Side players need high levels of sprint abilities to pass an opponent along the line, and STs need to be able to change directions more appropriately and pass an opponent to get into the penalty area [43].

Our study showed positive correlations between the AP and MRP indicators. As in the study by Bangsbo and Lindquist [23], we observed a correlation between the ${\dot{\text{V}}\text{O}}_{2\max}$ and TD, HSR distance, HIR distance, and SPR distance. Athletes with a higher ${\dot{\text{V}}\text{O}}_{2\max}$ have better lactate-removal capability and phosphocreatine regeneration [45] and therefore can better meet match demands [46] and recover faster between high-intensity actions [47, 48]. Conversely, the lack of correlation between the ${\dot{\text{V}}\text{O}}_{2\max}$ and MRP found by other authors [7, 8, 17, 24] was explained by the fact that a soccer match is intermittent in nature, and ${\dot{\text{V}}\text{O}}_{2\max}$ determines exercise capacity in continuous tests [3].

Similar to the report by Helgerud et al. [9], we observed a correlation between the AnT and MRP indicators. Modric et al. [8] reported that AnT correlated with HSR, SPR, and HIR distances but only among side players (FBs and wingers). Cairns [49] found that low AnT levels were associated with raised lactate and hydrogen ion (H^+^) concentrations, a decrease in intramuscular pH, and metabolic acidosis. Low AnT levels may therefore limit the MRP indicators related to the amount of energy consumed in low- (mitochondrial metabolic pathways) and high- (anaerobic metabolic pathways) intensity exercises.

We also observed positive correlations between the MAS and TD, HSR distance, and HIR distance. Correlations between performance in the MAS test (an indirect method of MAS determination) and the TD or HSR distance in elite male soccer players were reported only by Krustrup et al. [17] and Kalapotharakos [21]. The discrepancy in the results may be explained by the different ways in which MAS was determined. In our study, the MAS was measured using a direct laboratory ergospirometric method. Non-laboratory methods can underestimate or overestimate MAS due to differences in protocols (e.g., shuttle vs. straight-line running) and estimation of MAS based on the maximum load in the test and therefore at different ${\dot{\text{V}}\text{O}}_{2}$ values. Our study also showed positive correlations between the Yo-Yo IRT2 score and all MRP indicators. Previous studies have found similar relationships between the Yo-Yo IRT1 and Yo-Yo IE2 scores and TD and HIR distance [17, 18] and between the Yo-Yo IE2 score and TD, HSR distance, and HIR distance, but only in forwards and central defenders [27]. The correlation between the Yo-Yo test score and MRP indicators may be explained by the fact that the type of effort (intermittent shuttle running), distance covered per minute in the test, rest interval, and 180° directional change show a high specificity for the running demands in soccer [10, 17, 40].

The present in-depth analysis showed that AP was associated with MRP in the same way regardless of playing position and contextual factors. In detail, while controlling for playing position and match-related contextual factors, we found significant positive associations between the ${\dot{\text{V}}\text{O}}_{2\max}$ (Table 5), AnT (Table 6), MAS (Table 7), Yo-Yo IRT2 score (Table 8), and TD. Positive associations were also observed between the Yo-Yo IRT2 score and HSR and HIR distances (Table 8). This means that irrespective of playing position and contextual factors, a higher AP enables athletes to cover a greater TD during a match. For an increase in the ${\dot{\text{V}}\text{O}}_{2\max}$ by every 1 mL · kg−1 · min^−1^, AnT by every 1 km · h^−1^, MAS by every 1 km · h^−1^, and Yo-Yo IRT2 score by every 100 m, the TD increased on average by 1.920 (1.7%), 5.601 (5.1%), 5.419 (4.9%), and 1.900 (1.7%) m/min, respectively. In contrast, Kavanagh et al. [50], using the LMM but without statistical controls for playing position and contextual factors, found no statistically significant relationship between 1200 m shuttle test scores and distances covered at varying intensities in English Premier League matches. Furthermore, in our research, for every 100 m increase in the Yo-Yo IRT2 score, the HSR distance increased on average by 0.4 m/min (6.2%) and HIR distance by 0.5 m/min (5.9%). Based on standardized coefficients, the strongest predictors for TD were ${\dot{\text{V}}\text{O}}_{2\max}$ and AnT (large effect size), while for HSR distance and HIR distance the only significant predictor was Yo-Yo IRT2 score (medium effect size). For SPR, none of the AP predictors achieved statistical significance. A potential reason for such results is that at the highest sporting level in soccer (the players studied play for a club that is a multiple national champion and regularly participates in European cups), positions such as CMs and FBs, which are characterized by the highest requirements in terms of MRP, are played by selected players in terms of technical and tactical skills and AP level. Players who, due to a lower AP level, are unable to meet the match requirements of these positions are moved to other positions in the early training process (youth soccer academies) or are discarded at the scouting or pre-transfer exercise test stage. Additionally, due to the characteristics of the match effort and tactical tasks (strong positional play with good head play, frequent but short sprint efforts, and the highest number of jumps), CBs or STs are often taller and heavier than other players, which may affect the lower AP level [42, 43]. Due to the individualization of training, the higher AP values in midfielders and FBs can probably be attributed to the higher amount of aerobic training than in CBs and STs [43]. Finally, programming training loads based on AP results in athletes with a higher AnT or MAS (midfielders and FBs) performing training at a higher absolute intensity, which can further increase the observed differences. In the current study, the CMs and FBs had a higher AP and therefore MRP than the WMs, STs, and CBs. The above-noted relationship is not affected by the fact that contextual factors are systematically associated with MRP. Regardless of playing position, the soccer players studied had significantly higher MRP indicators in home matches than in away matches (TD: 2.5 m/min, HSR distance: 0.4 m/min, SPR distance: 0.24 m/min, and HIR distance: 0.65 m/min) and in European cups than in domestic leagues (TD: 3.0 m/min, HSR distance: 0.42 m/min).

### Limitations

Despite the findings of the current study, there are some limitations to be considered. The main limitation of our research is the fact that we included only one soccer team that presented the highest possible level in the national competition, which is also related to the small sample size and, at the same time, the limited sample size of the individual playing positions. This limits the generalizability of our results to teams with a lower sporting level, indicating the need for additional analysis and cautious interpretation. To overcome this limitation, further studies should consider including more soccer teams with different performance levels and various tactical formations. Another limitation is that, as in many previous studies [7, 8, 17, 24], AP was tested only at the beginning of the preseason and not after the end of it or even during the whole season [51]. Finally, other contextual factors, such as match score, opponent level, or opponent team formation, were not considered.

## CONCLUSIONS

Despite individual variability of soccer players, AP is related to MRP independently of playing position and contextual factors. Higher values of all measured AP indicators are positively associated with the TD covered during a match. Additionally, a higher Yo-Yo IRT2 score is also positively related to the HSR and HIR distances. The strongest predictors for TD were ${\dot{\text{V}}\text{O}}_{2\max}$ and AnT.

### Practical Applications

This research allows practitioners to identify physical match outputs using the selected AP indicators. Players characterized by a higher level of AP showed a greater MRP. This may significantly impact training strategies. Knowing the pre-season AP level of soccer players allows a certain degree of prediction of their on-field performance during the season. Additionally, by knowing the AP values of individual players before the season, conditioning coaches, in order to obtain the appropriate AP level for each playing position, can individualize training, taking into account the requirements for soccer players in each position.

Finally, knowing the predictive power of individual AP indicators can influence testing strategies in the pre-season period. A better understanding of the relationship between individual AP indicators and MRP will allow coaches to select exercise tests appropriate for their conditioning philosophy and playing strategy.

In summary, this study emphasized the value of integrating AP metrics into individualized training and player role management in elite soccer. However, the findings should be interpreted with caution due to the limited sample. Future research should involve broader and more diverse team samples, assess AP at multiple time points throughout the season, and account for additional contextual influences to further refine understanding of the relationship between AP and MRP.
